# Immune response accelerated telomere shortening during early life stage of a passerine bird, the blue tit (*Cyanistes caeruleus*)

**DOI:** 10.1098/rsbl.2024.0618

**Published:** 2025-01-22

**Authors:** Matteo Schiavinato, Shivani Ronanki, Ignacio Miro Estruch, Nico van den Brink

**Affiliations:** ^1^ Cluster of Biomolecular Science, Division of Toxicology, Wageningen University and Research, 6708 WE Wageningen, The Netherlands

**Keywords:** telomere, virus, immune system, poly I:C, reactive oxygen species, blue tit

## Abstract

Dealing with infections is a daily challenge for wild animals. Empirical data show an increase in reactive oxygen species (ROS) production during immune response. This could have consequences on telomere length, the end parts of linear chromosomes, commonly used as proxy for good health and ageing. Telomere length dynamics may reflect the costs associated with physiological responses. In this study, immune system of blue tit (*Cyanistes caeruleus*) nestlings was experimentally challenged through a polyinosinic:polycytidylic acid (poly I:C) injection, a synthetic double-stranded RNA that mimics a virus, activating the pathway of immune response triggered via the toll-like receptors 3. This path is known to form ROS downstream. Immune response was quantified by white cell counts in blood, while brain lipoperoxidation has been evaluated as an indicator of oxidative damage. Finally, individuals’ telomere length shortening between days 8 and 15 after hatching was measured in erythrocytes. Challenged nestlings showed increased leukocyte number when compared with control (treated with a saline solution), lower brain lipid peroxidation (likely as a result of a compensatory mechanism after oxidative stress burst) and accelerated telomere shortening. These findings support the ‘ageing cost of infections pathway’ hypothesis, which supposes a role for infections in quick biological ageing.

## 1. Introduction

Telomeres are parts of genomic DNA at the end of linear chromosomes, made of repeated sequence (TTAGGG)n in vertebrates [1]. They play an important role in chromosome stability, protecting them from being detected as DNA double-strand breaks, stopping degradation and fusion with other chromosome ends [2]. Telomere length decreases progressively according to cell divisions, due to incomplete replication of the DNA end during lagging strand DNA synthesis by polymerases (enzymes that catalyse the synthesis of DNA), a mechanism called the ‘end replication problem’ [3]. Another mechanism by which telomeres may shorten is via oxidative stress [4], where the level of reactive oxygen species (ROS) is higher than the capacity of antioxidant defence, thus leading to excessive ROS exposure [5]. ROS can be generated from exogenous sources such as pollutants [6], or endogenous factors like mitochondrial metabolism [7]. Telomeres are especially sensitive to oxidative damage [8], because ROS specifically target their guanine bases forming DNA lesions [9]. These base modifications can initiate DNA base excision repair (BER) pathways by DNA glycosylase, an enzyme that catalyses the removal of damaged nucleotides [10] and thereby parts of the telomere. Although telomere shortening seems to be a general rule, also telomere lengthening has been observed [11], for example, in red blood cells of house sparrows (*Passer domesticus*) [12], Magellanic penguins (*Spheniscus magellanicus*) [13], Seychelles warblers (*Acrocephalus sechellensis*) [14] and humans (*Homo sapiens*) [15] and in buccal mucosa cells of edible dormice (*Glis glis*) [16]. Lengthening may occur because of the activity of telomerase enzyme (which maintains telomere length by adding new sequences) [17], high turnover in hematopoietic cells [18] or other reasons yet to be clarified. Observations of both telomere shortening and lengthening during ageing suggest we should rethink telomere length as a dynamic length that reflects the current individual’s state and its physiological trade-offs, rather than a progressive and unstoppable shortening according to growth. For instance, malaria infection by *Plasmodium falciparum* increased telomere shortening in blood cells during the first three months after infection in humans [15]. However, following infection, CDKN2A expression (a gene linked to cell senescence) declined, resulting in increasing telomerase activity that gradually restored the telomere length to pre-infection levels over 1 year. Therefore, although telomere length has become a biomarker to estimate health status [19], telomere length dynamics may better underline processes related to interactions between individuals and their physiological challenges, as well as their future life-history strategies and adaptive responses [20].

The effect of environmental stressors on telomere length could be particularly important during early life stages, when telomere loss is already strongly pronounced [21] and when animals are more vulnerable. Early life exposure to pathogens can be a challenge, particularly in natural bird populations and in nestlings without the protection of feathers, which are susceptible to infection, e.g. from vectors such as mosquitoes [21] or ticks [22]. Parasites and infections may trigger the immune system, increasing telomere shortening through the direct cost of immune activation and reduced investment in tissue maintenance [22]. Furthermore, an increase in ROS is a feature of many viral infections and can be caused by direct effects of the virus on cells and indirect effects of inflammatory responses [23]. Therefore, infections may represent an important stressor that could increase telomere shortening.

In this study, the effect of immune response on telomere length dynamics has been addressed: blue tit (*Cyanistes caeruleus*) nestlings were experimentally challenged using polyinosinic:polycytidylic acid (poly I:C) as a model compound to mimic a viral infection. Poly I:C is a double-stranded RNA similar to some viruses, which is recognized by the toll-like receptors 3 (TLR3) of macrophages, lymphocytes and dendritic cells, inducing interferon α and β as responses [24] and the generation of ROS for phosphorylation and nuclear translocation of STAT1 and STAT2 [25], key components of the transcription factor complex in interferon signalling pathways [26]. The TLR3, involved in the detection of pathogens [27], is also present in many other cells, such as epithelial cells [28] and erythrocytes [29]. We hypothesize that the activation of the immune system through a simulated infection leads to the generation of ROS and accelerates telomere shortening in exposed birds.

## 2. Methods

### (a) Routine in field

The study was conducted over the spring of 2022. Wild pairs of blue tits (*C. caeruleus*) freely breed in artificial nest boxes in Wageningen (51.981063, 5.636866) and Rhenen (51.973095, 5.577511) in The Netherlands. The field sites count 100 nest boxes installed in 2021 along roads, about 3 m in height, in oak trees (*Quercus robur*). From the middle of March, the boxes were routinely checked to monitor the stage of breeding. In total, 32 nestlings from eight different nests were included in the experiment. At day 8 post-hatching (hatching day as ‘day 1’), chicks’ nails were coloured with nail polish to recognize individuals during development. Morphometric measures (mass, tarsus and wing length) were collected at day 8, and subsequently, 40 μl of blood was sampled from the brachial vein using 25 G needles (Sterican^®^) and 75 μl sodium-heparinized capillary tubes (VITREX^®^). Blood samples were centrifuged (microcentrifuge VWR^®^ MiniStar silverline) for 10 min at 2000*g* to separate plasma from red blood cells, and samples were stored at −80°C. On day 13 after hatch, two random chicks from each nest were challenged with an intra-muscular injection of 10 μl of poly I:C (Sigma Aldrich), and two others received a saline solution as control, using sterile syringes with 27 G needles (Sterican^®^). Therefore, four animals in each nest were used for this study. Poly I:C salt was diluted in saline water and injected into the pectoral muscle with a dose of 2 μg kg^−1^ body weight. This value has been chosen according to previous studies on house sparrows (*Passer domesticus*) [30] and barnacle goslings (*Branta leucopsis*) [31], which have demonstrated that this dose is not lethal but is capable of triggering an immune response. Poly I:C solution was prepared fresh every day by resuspending the salt in sterile saline at a concentration of 2 mg ml^−1^, until complete solubilization. At day 15, the day after hatching, the blood sampling procedure was repeated following the same steps as on day 8. In addition, a blood smear for each individual was collected on a microscope slide (VWR^®^, 631-1552). At 15 days of life, the animals in the study were euthanized as part of shared experiments, applying the principle of reduction in animal testing. Tissues were collected for analysis and stored at −80°C.

### (b) Telomere length estimate

DNA was extracted from 5 μl of red blood cells using DNeasy^®^ Blood & Tissue Mini Spin Columns (Qiagen), following the manufacturer’s instructions. DNA quantity and purity were measured using a NanoDrop-One spectrophotometer (ThermoScientific). Telomere length was measured by quantitative real-time amplification method (qPCR) [32] with GAPDH as the control gene [33]. The qPCR was performed using a Rotor-Gene Q real-time PCR cycler (Qiagen), using 5 ng of DNA with 1 μl of each primer (forward and reverse) at a concentration of 10 μM, with 10 µl of QuantiFast mix with SYBR green, in a final reaction volume of 20 μl. The following primers were used: telomere forward tel1b (5′-CGGTTTGTTTGGGTTTGGGTTTGGGTTTGGGTTTGGGTT-3′) and reverse tel2b (5′-GGCTTGCCTTACCCTTACCCTTACCCTTACCCTTACCCT-3′); GAPDH forward (5′-TGTGATTTCAATGGTGACAGC-3′) and GAPDH reverse (5′-AGCTTGACAAAATGGTCGTTC-3′). These sets of primers have been shown to be applicable for blue tit [34]. Reactions were performed in duplicate and the mean was used, ensuring the difference in *Ct* values between duplicates was equal to or less than 0.3 [35]. Samples at day 8 and at day 15 of the same individuals were distributed in the same runs. Each nest (control and treatment birds) was included in the same run, as well as the GAPDH and telomere reactions for the same individual. The qPCR profile was the following: holding at 95°C for 5 min, first cycle at 95°C for 10 s, then 60°C for 15 s, followed by 60 s at 72°C. Finally, melt phase ramped from 72°C to 95°C rising by 1°C each step, waiting 90 s of pre-melt conditioning on first step and waiting 5 s for each step afterwards. On each run, a standard curve made by 6 serial double dilutions from 2 to 0.06 ng of the total pool of DNA samples has been included (plus a negative control), for both GAPDH and telomeres. Efficiencies of standard curves were within 0.90 and 1.10 for both GAPDH and telomeres. Telomere length was calculated as *T*/*S* ratio, which is the standard used in qPCR-based telomere studies [36]. Amounts of telomeres and the single-copy gene (GAPDH) sequences were calculated with respect to their standard curves based on the *Ct* values of qPCR reactions [37]. Telomere length dynamics were quantified in % as the variation of the ratio (*T*/*S*) of telomere tandem copy repeat number (*T*) to GAPDH as a single gene copy number (*S*) from day 8 to day 15: {[(*T*/*S*)_15_ − (*T*/*S*)_8_]/(*T*/*S*)_8_} × 100.

### (c) Immune response quantification

Blood smears were stained with Hemacolor Rapid staining (Sigma-Aldrich, Zwijndrecht, The Netherlands) and counted using a light microscope at 1000× magnification with immersion oil (Zeiss, Jena, Germany). The number of leukocytes and red blood cells was counted until the vision contained the 5000th red blood cell [31]. Heterophils, eosinophils, basophils, monocytes and lymphocytes were included in leukocyte counts, identified according to their morphological characteristics [38]. The percentage of leukocyte populations relative to erythrocytes was calculated for each animal as {[(no. leukocytes) − (no. erythrocytes)]/(no. erythrocytes)} × 100.

### (d) Oxidative stress assay

Whole brain lipoperoxidation was used as an oxidative stress biomarker [39]. The Lipotiss test (Diacron International, Grosseto, Italy) was used to assess lipoperoxidation. The method is based on the ability of peroxide to promote the oxidation of Fe²^+^ to Fe³^+^. The produced ion binds to thiocyanate, developing a pink color complex that is photometrically measurable. Procedures followed the manufacturer’s instructions with minor adjustment according to Terraneo *et al*. [40]: 200 mg of pre-mixed tissue was homogenized in 0.5 ml of Milli-Q water, centrifuged for 5 min at 15 000*g* and washed three times with Milli-Q water. After removing the supernatant, 500 μl of R1 reagent (mixture of indicators) was added, and vials were mixed and centrifuged for 5 min at 1400*g*. Afterwards, 250 μl of supernatant was added to a 96-well plate (Cellstar^®^, 655-180) together with a standard curve (plus a blank) made of four concentrations of 4000 µEq l^−1^ terbutylhydroperoxide diluted in 250 μl of reagent R1. To the solutions, 10 μl of reagent R2 (ferrous ions solution) diluted in a ratio of 1 : 4 with reagent R1 was added. After 5 min of incubation at 37°C, the optical density was read at λ = 505 nm using a spectrophotometer (Tecan, Infinite-M nano^+^). The concentration of lipoperoxides was calculated and expressed as nanoequivalents of hydroperoxides (ROOH) per gram of tissue. All individuals in the study were analysed on the same single plate.

### (e) Statistical analysis

Statistical analyses were conducted in R (v. 4.3.1). To compare immune response between control and treatment groups, a linear mixed effects model was fitted using the ratio of leukocytes to erythrocytes as response variable [41], and type of treatment (poly I:C or saline) plus body condition at day 15 as independent variables. Individual’s body condition was calculated by dividing body mass by tarsus length cubed [42]; this is a common measure of energy reserves and health status in birds, representing animal mass corrected for structural size [43]. To investigate if immune response led to a change in oxidative state, brain lipid peroxidation level was used as dependent variable in a new model, while the ratio between the number of leukocytes and erythrocytes, and body condition as independent variables. Finally, to examine whether poly I:C challenge affected telomere shortening between days 8 and 15, a new linear mixed model was used, with the telomere shortening rate as dependent variable. Treatment, growth in mass between days 8 and 15 (calculated as: [(Mass_15 − Mass_8)/(Mass_8)] × 100) and body condition at day 15 have been set as independent variables. Growth was included because, according to theoretical models, as cell divisions increase telomere length decreases [44]. In all models, nest of origin was used as a random effect. The ‘lmer’ function in ‘lme4’ package [45] was used to build the models, while, to test the significance of the main predictor variables, we used the ‘lmerTest’ package [46]. Significance was taken at *α* = 0.05. The hypothesis of homoscedasticity has been tested and confirmed with an *F*-test of equality of variances and the assumption of normal distribution of the model’s residual with a Shapiro–Wilk normality test.

## 3. Results

**Figure 1.**
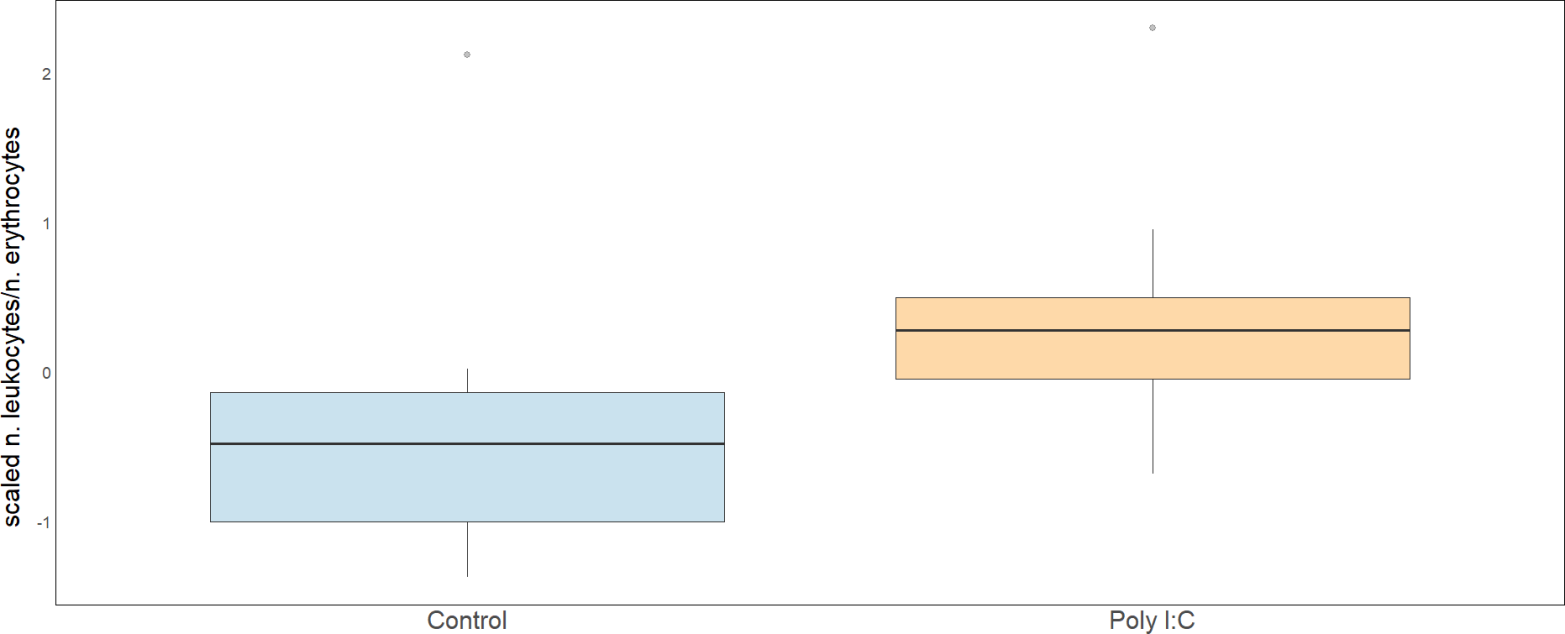
Boxplots of scaled number of leukocytes compared with erythrocytes at day 15 for control and treatment.

The results of the first model revealed that poly I:C treatment significantly increased the number of leukocytes compared with the control (table 1 and figure 1). This finding supports the hypothesis of immune response after treatment. Body condition at day 15 had no significant effect on the ratio of leukocytes to erythrocytes, indicating that the immune response was primarily driven by treatment rather than the animal’s physical condition.

**Table 1.** Model estimates for the effect of treatment on the leukocytes/erythrocytes ratio.

predictors	estimates	CI	*p*
intercept	−2.13	−8.28 to 4.02	0.465
treatment (poly I:C)	0.68	0.09–1.28	**0.028**
body condition at day 15	0.85	−1.98 to 3.68	0.526

Values of *p* < 0.05 are indicated in bold. Predictors are scaled.

**Figure 2.**
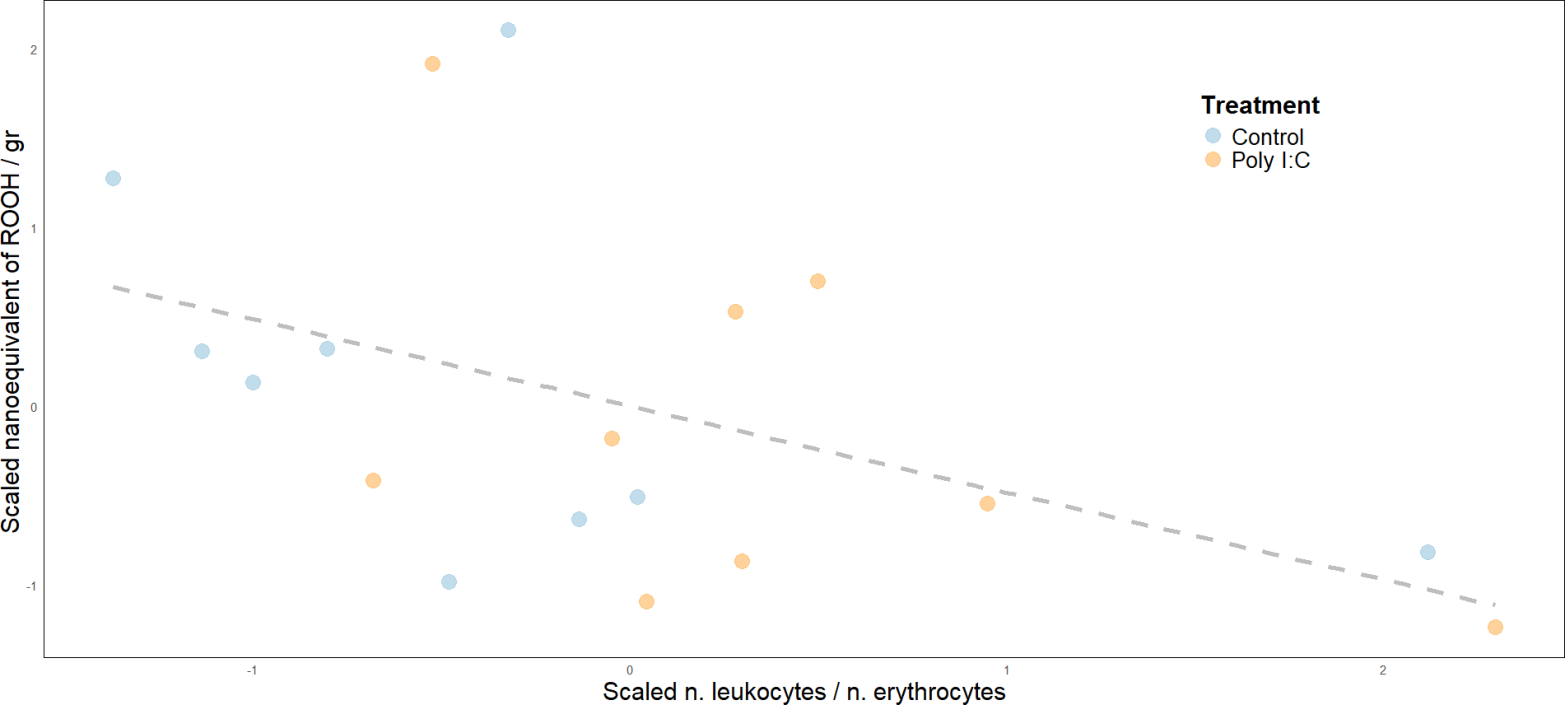
Overall regression between brain lipoperoxidation at day 15 and immune response estimated as the number of leukocytes compared with erythrocytes in blood smears at day 15.

The following model evaluated brain lipoperoxidation as a function of immune response. The output (table 2) showed a marginally negative relationship between leukocyte number and lipoperoxidation. Through a stepwise approach that removed body condition as an independent variable (not statistically significant), the outcome of the simplified model highlighted a negative covariation between the number of leukocytes in blood and brain lipoperoxidation. This trend suggests how in our dataset, a higher immune response is associated with lower oxidative damage (figure 2).

**Table 2.** Model estimates of the effect of leukocytes/erythrocytes ratio on lipoperoxidation.

predictors	estimates	CI	*p*
intercept	1.01	−0.20 to 2.22	0.095
white cell proportion	−0.97	−1.99 to 0.05	0.062
body condition at day 15	0.01	−0.37 to 0.39	0.953
**simplified model without accounting for body conditions**			
intercept	0.08	−0.48 to 0.31	0.653
white cell proportion	−0.40	−0.80 to 0.01	**0.046**

Values of *p* < 0.05 are indicated in bold. Predictors are scaled.

**Figure 3.**
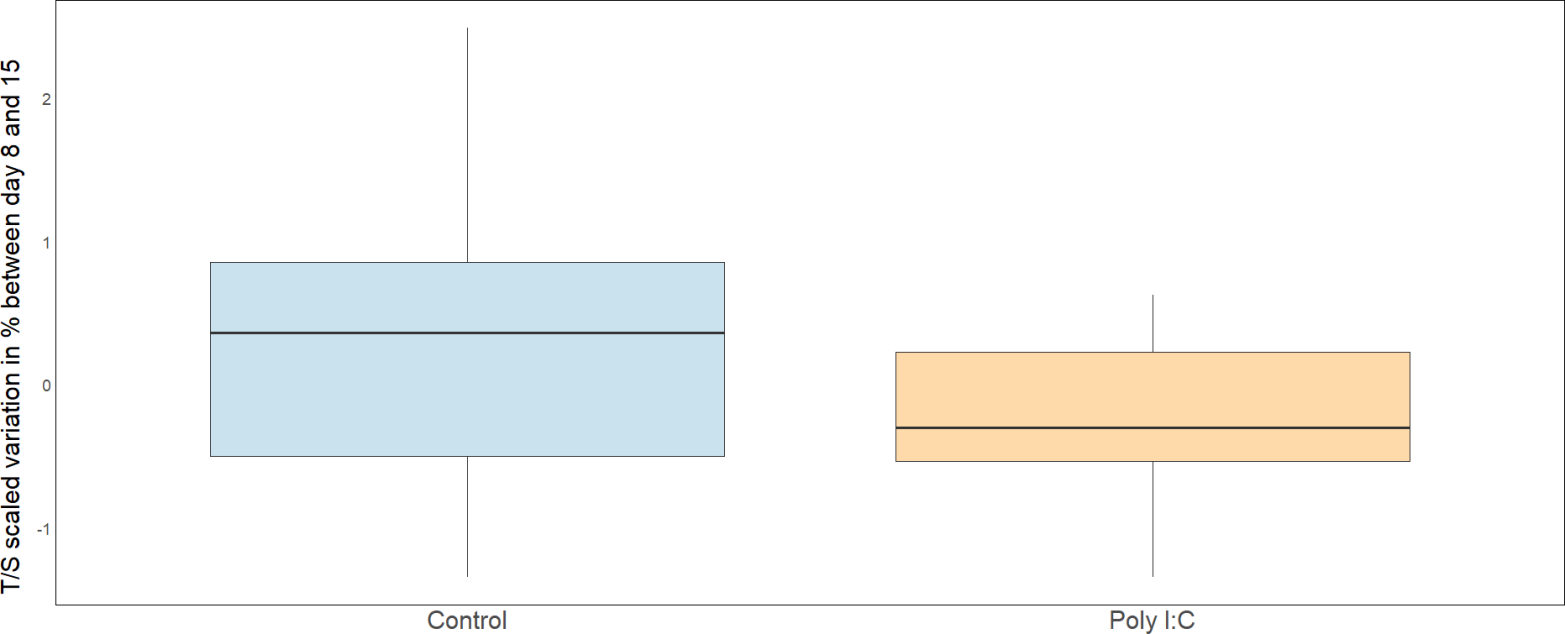
Boxplots of scaled telomere length variations in per cent between days 8 and 15. Values greater than 0 correspond to telomere lengthening, negative values to shortening.

The third model evaluated the effect of treatment on telomere length dynamics between day 8 and day 15. Challenged animals experienced a more pronounced reduction in telomere length compared to control (table 3 and figure 3). Body condition at day 15 and growth mass did not influence telomere lengths.

**Table 3.** Model estimates of the effect of treatment on blue tit telomere length dynamics.

predictors	estimates	CI	*p*
intercept	0.41	−0.09 to 0.91	0.102
treatment (poly I:C)	−0.79	−1.53 to 0.06	**0.036**
growth	0.04	−0.35 to 0.43	0.837
body condition at day 15	−0.21	−0.60 to 0.19	0.286

Values of *p* < 0.05 are indicated in bold. Predictors are scaled.

## 4. Discussion

This study revealed how the activation of the immune system through the viral-like compound poly I:C could have had an impact on telomere length of blue tit nestlings. In fact, telomere shortening in challenged chicks was significantly accelerated when compared with siblings in control.

First, we validated if treatment triggered the immune system as expected, since poly I:C induces an innate immune response [47] promoting activation of peripheral blood leukocytes [48]. This has been proved by comparing the number of leukocytes between treatment and control animals after challenge. In our study, treatment increased leukocyte numbers, indicating immune response.

Second, we investigated whether the immune response increased oxidative stress. This has been previously reported [49], as well as apoptotic cell death through formation of ROS poly I:C exposure [50]. Indeed, poly I:C RNA binds the TLR3 receptor involved in antiviral and inflammatory responses, leading to ROS generation needed for activation of macrophages’ immune responses [25]. In the current study, brain lipoperoxidation was used as an oxidative stress biomarker. Free radicals, in fact, bind membrane lipids causing peroxidation and generation of highly reactive and electrophilic unsaturated aldehydes, such as malondialdehyde (MDA) and 4-hydroxynonenal (HNE) [51]. The brain, with its high oxygen consumption and lipid-rich content, is very susceptible to oxidative stress damage [52]. In fact, it has the second highest lipid content after adipose tissue [53]. Previous studies have shown how poly I:C in rats increased brain lipid peroxidation [54]. Opposite to what we expected, we found a negative relationship between immune response and lipoperoxidation: this was lower when the number of leukocytes in blood was higher. Nevertheless, in a study published by He *et al.*, fish (*Scophthalmus maximus*) fed with poly I:C had lower liver MDA content than control [55], which would suggest treatment triggered also anti-oxidative-stress activity. We hypothesize, therefore, that poly I:C could have initiated an immune response with consequent increased oxidative stress. Subsequently, by the time we measured lipoperoxidation levels in the brain, compensatory mechanisms might already have been activated to mitigate further damage. In the brain, astrocytes provide antioxidant support to neurons through the Nrf2 pathway and regulation of a cohort of antioxidant genes; furthermore, many neuronal antioxidant genes enabling increases in ROS are knocked down by the enhanced antioxidant capacity of both glutathione and thioredoxin–peroxiredoxin systems [56]. These homeostatic mechanisms, therefore, may have been activated after the oxidative stress burst because of treatment, enhancing antioxidant or repair processes and reducing lipoperoxidation 2 days after exposure. Finally, we established a poly I:C effect on telomere length dynamics: the treatment group showed higher telomere shortening compared with control. Previous evidence suggests that viral infections are associated with telomere attrition; however, the degree to which any associations are causal remains unclear [57]. Interestingly, a previous study found no association between herpesvirus infection and telomere shortening in magnificent frigatebird (*Fregata magnificens*) nestlings [58]. Authors, however, found no variation in nitric oxide in infected birds, and so herpesvirus infection may not activate pathways that would trigger an increase in ROS, leading then to telomere shortening. The researchers hypothesized that the lower metabolic rate due to the slow growth of frigatebirds might have masked the effects of infection on telomeres. In this view, the supposed high metabolism of our blue tit nestlings [59] may have accelerated the effect of infection on telomeres. In addition, observations on siskins (*Spinus spinus*) [60] and great reed warblers (*Acrocephalus arundinaceus*) [36] suffering from malaria showed a reduction in telomere length in erythrocytes compared to uninfected conspecifics, and malaria seems to be associated with ROS increasing [61]. In fact, inflammation exacerbates the rate of telomere attrition, leading to telomere dysfunction and accelerating the cellular ageing process [62].

Generally, oxidative stress might contribute to telomere shortening, as supported by a meta-analysis by Armstrong & Boonekamp [63]. However, as commented by Reichert & Stier in their review [64], our understanding of this link remains incomplete. Indeed, in our study, although telomere length dynamics were measured over 7 days, the simulated infection had an effect only over 2 days. Given this very short time for oxidative stress to act on telomeres, we question to what extent shortening has been a consequence of ROS or to an increase in blood leukocytes, whose rapid division as an immune response might lead to shortening of their telomeres. However, in avian blood, a normal haematocrit ranges from 40% to 60%, and the general ratio of red blood cells to white blood cells is around 100 erythrocytes to 1 leukocyte [65]. Since avian erythrocytes are nucleated, the majority of DNA extracted from blood originates from them; therefore, even minor changes in leukocyte proportion because of infections should have little impact on average telomere length of blood DNA. However, this hypothesis should not be underestimated, since we do not know the extension of the increase of white blood cells after poly I:C challenge. Anyhow, a correlative analysis in our dataset shows a lack of covariation between the number of leukocytes and telomere length variation between days 8 and 15 (*r* = −0.02, *r*² = 0.00, *p* = 0.913), which suggests no impact of changes in blood cell population on telomere dynamics. Although the mechanism leading to immune response to telomere shortening is still unclear, this study might support the ‘ageing cost of infections pathway’ hypothesis suggested by Giraudeau *et al*. [22], which states that infections may lead to faster biological ageing. Our findings suggest that immune responses may accelerate telomere shortening, and that causative factors could be the production of ROS downstream from the binding of the receptor TLR3 and the high metabolism of blue tit nestlings. Since nestlings’ telomere shortening has been shown to reflect individual survival probability to adulthood [66], this biomarker could underline the current individual health state and trade-offs between immune responses to infection and premature biological senescence. To validate this *post hoc* observation we described, we encourage further studies on how telomere length dynamics are altered by the experimental activation of the immune system.

## Data Availability

Data, metadata and code are provided in the electronic supplementary material [67].
